# Serum Galectin-3 Levels Predict Recurrences after Ablation of Atrial Fibrillation

**DOI:** 10.1038/srep34357

**Published:** 2016-09-28

**Authors:** Nicolas Clementy, Nazih Benhenda, Eric Piver, Bertrand Pierre, Anne Bernard, Laurent Fauchier, Jean-Christophe Pages, Dominique Babuty

**Affiliations:** 1 Cardiology Department, Trousseau Hospital, François Rabelais University, Tours, France; 2 Biochemistry Department, Trousseau Hospital, François Rabelais University, Tours, France

## Abstract

Galectin-3 is a biomarker of fibrosis and atrial remodeling, involved in the mechanisms of initiation and maintenance of atrial fibrillation (AF). We sought to study the accuracy of galectin-3 level in predicting recurrences of AF after ablation. Serum concentrations of galectin-3 were determined in a consecutive series of patients addressed for AF ablation in our center. After a 3-month blanking period, recurrences of atrial arrhythmias were collected during the first year in all patients, using Holter monitoring at 3, 6 months and 12 months. A total of 160 patients were included, with a mean galectin-3 rate was 14.4 ± 5.6 ng/mL. At 12-month, 55 patients (34%) had reexperienced sustained atrial arrhythmia. Only higher galectin-3 level (HR = 1.07 [1.01–1.12], p = 0.02) and larger left atrial diameter (HR = 1.07 [1.03–1.12], p = 0.001) independently predicted recurrence. Patients with both galectin-3 level <15 ng/mL and left atrial diameter <40 millimeters had a 1-year arrhythmia-free survival rate − after a single procedure without anti-arrhythmic drug − of 91%, as compared with 41% in patients with galectin-3 ≥ 15 and left trial diameter ≥40 (p < 0.0001), whether AF was paroxysmal or persistent. Galectin-3 and left atrial diameters, rather than clinical presentation of AF, predict recurrences after ablation.

Ablation of atrial fibrillation (AF) is a recommended therapy in symptomatic patients, when appropriate^1^. It is however associated with different outcomes depending on the clinical presentation of the disease, with a higher rate of recurrence after ablation in patients with persistent AF (Ps-AF) than in those with paroxysmal AF (Px-AF)^2^. Left atrial remodeling, structural, electrical or autonomic, is known to be associated with the severity of the disease^3^. Specifically, structural remodeling with progressive fibrosis extension within the left atrium (LA), favors the development of focal activities and reentries, initiating and maintaining AF^4^. Recently, galectin-3 (Gal-3), a member of the ß-galactoside-binding lectins family, has been suspected of playing a role in promoting atrium fibrosis in patients with AF^3^. Gal-3 levels are significantly higher in patients with Ps-AF, and have also been correlated with structural LA remodeling assessed with delayed enhancement on magnetic resonance imaging (MRI)^5^,^6^.

We then hypothesized that higher levels of Gal-3, reflecting a more extensive structural LA remodeling, and thus a more advanced progressive disease, could predict recurrences of arrhythmia after AF ablation.

## Methods

### Inclusion

Consecutive patients with symptomatic AF referred to our department for ablation between February 2013 and May 2014 were included in this study. Exclusion criteria included all non-cardiac conditions with expected elevated Gal-3 levels, such as liver cirrhosis, pancreatitis or chronic inflammatory disease^7^. Patients with a prior ablation for AF were also excluded. Collected clinical data included symptoms (EHRA classification) and history of arrhythmia, presence of thrombo-embolic risk factors (according to CHADS_2_ and CHA_2_DS_2_-VaSC scores), and past and current medications. Trans-thoracic echocardiography and cardiac magnetic resonance imaging or CT-scan were performed to assess left ventricular function and LA diameter and volume before ablation.

All methods, including the ablation procedure, were carried out in accordance with the guidelines^1^. The ethics committee for human research of the University Hospital Center of Tours (France) approved the study protocol. All patients signed informed consent before inclusion.

### Galectin-3

During the early stage of the AF ablation procedure, a blood sample was collected peripherally through the femoral vein sheath in order to determine the anticoagulation time. Measurement of serum Gal-3 level was performed on residual samples.

Determination of the Gal-3 level was completed using the VIDAS Galectin-3 kit (bioMérieux, Marcy-l′Etoile, France). VIDAS Galectin-3 is an automated quantitative test. The kit measuring range is 3.3–100 ng/mL. The assay principle is a one-step immunoassay sandwich method with final fluorescent detection, and has been previously validated in heart failure patients^8^.

### Ablation

Procedures were performed under general anesthesia, with an objective for anticoagulation time of 300 seconds. A 4-millimeter irrigated-tip catheter was used in all patients to deliver radiofrequency energy (Thermocool SF, Biosense Webster; Flexability, St Jude Medical). A circular lasso catheter (Biosense Webster, St Jude Medical) was used for mapping. After transeptal puncture, antral pulmonary vein isolation (PVI) was performed in all patients. A bidirectionnel block was systematically obtained in all veins.

In patients with Ps-AF and remaining arrhythmia at that stage a stepwise approach was performed: sequentially, anterior roof and mitral isthmus lines were obtained (endocardially, and epicardially through the coronary sinus, when necessary), complex fractionated atrial electrograms (CFAEs) within left atrium (left atrial appendage, inferior-posterior wall, interatrial septum), coronary sinus, and right atrium (crista terminalis, superior venacava, cavotricuspid isthmus), were mapped and defragmented when necessary. Stable atrial tachycardia were systematically mapped and ablated. When return to sinus rhythm was obtained, either with ablation, or with electrical cardioversion at the end of procedure, bidirectional block was confirmed on all performed lines.

### Follow-up

Recurrence was defined as ≥1 documented sustained episode (≥30 seconds) of any atrial arrhythmia, symptomatic or not, on any ECG or Holter monitoring strip (scheduled or additional), after a single ablation procedure, after a 3-month blanking period.

During the blanking period, antiarrhythmic drugs were continued in most of the patients, and a cardioversion was performed in the event of persistent recurrence. At the end of the blanking period, antiarrhythmic drugs were systematically discontinued in all patients. All patients were rigorously followed for 12 months. A 24-hour Holter monitoring was performed at 3 and 6 months along with a clinical examination and a resting ECG. A 7-day Holter recording was systematically performed at 12 months (Spiderview, Sorin Group, Le Plessis-Robinson, France).

### Statistical analyses

Analyses were performed using JMP software version 9.0 (SAS Institute Inc., Cary, NC, USA). Numeric data were expressed as mean ± standard deviation (95% confidence interval). Student T-test and Chi-2 were performed for comparison between groups. A Cox proportional hazard model was used to assess the factors independently associated with recurrence. The main confounding factors were tested in univariable analysis, and parameters significantly associated with recurrence (p-value < 0.05) were used for analyses in the multivariable model. Parameters derived from continuous numerical variables were determined using receiver operating characteristic (ROC) curves analysis to obtain accurate cutoff values. Survival curves were calculated using the Kaplan-Meier method, and a log-rank test was used to evaluate overall differences between groups. A p-value < 0.05 was considered significant.

## Results

### Population

One hundred and sixty patients met the criteria for inclusion in the study, of whom 88 with Px-AF (55%). Patients were predominantly men (71%), with a mean age of 61 ± 10 years. Hypertension was found in 49% of patients, and diabetes in 17%; 15% had a CHADS_2_ score >2, 26% a CHA_2_DS_2-_VASC score >2. Mean ejection fraction was 54 ± 11%, LA diameter (LAD) 42 ± 8 mm, and mean Gal-3 level 14.5 ± 5.5 ng/mL. All characteristics of the patients are reported in Table 1.

### Ablation

Pulmonary veins isolation was performed in all patients (bidirectional block 100%).

In Ps-AF patients, the roofline was ablated in 93% of patients (bidirectional block 100%), the mitral isthmus line in 83% (bidirectional block 95%), CFAE ablation in 61%, and a cavotricuspid isthmus line in 22% (bidirectional block 100%). A return to sinus rhythm during ablation, without cardioversion, was achieved in 49% of the Ps-AF patients.

### Recurrences

No patient was lost during follow-up. Sixteen patients (10%) were in persistent atrial arrhythmia at the end of the blanking period. At 12 months, after a single procedure, 55 patients (34%) had re-experienced ≥1 documented sustained atrial arrhythmia: 23% of Px-AF and 49% of Ps-AF patients (p = 0.0006). Patients with recurrence were older, more likely to have Ps-AF, heart failure (HF) and hypertension, and had higher Gal-3 levels and larger LA (Table 1).

### Predictive Factors

In univariate analysis, serum Gal-3 level, hypertension, HF, Ps-AF, age and LAD were predictors of recurrence. In multivariable analyses, only Gal-3 level (HR = 1.07 [1.01–1.12] per-unit increase) and LAD (HR = 1.07 [1.03–1.12] per-unit increase) were independent predictors of recurrence (Table 2).

Using ROC curves analyses, an LAD ≥40 millimeters and a Gal-3 level ≥15 ng/mL were found to be accurate independent risk factors of recurrence at 1 year. Combining these two markers further improved accuracy. C-Statistics are reported in Table 3.

Baseline Gal-3 levels were significantly higher in patients with more risk factors, whatever the type of AF, paroxysmal or persistent (Table 4).

### Type of AF

In the subgroup of patients with paroxysmal AF (N = 88), after multivariable analyses, Gal-3 level was the only independent predictor of recurrences (HR = 1.13 [1.04–1.22] per unit increase, p = 0.004), LAD not being significant (HR = 1.05 [0.98–1.14] per unit increase, p = 0.15). Conversely, in the subgroup of patients with persistent AF (N = 72), after multivariable analyses, LAD was the only independent predictor of recurrences (HR = 1.08 [1.02–1.14] per unit increase, p = 0.004), Gal-3 level not being significant (HR = 1.03 [0.97–1.07] per unit increase, p = 0.46).

Baseline Gal-3 levels were found to be similar in Px-AF and Ps-AF patients with an identical number of risk factors (Table 4). Px-AF patients with 2 risk factors had significantly higher Gal-3 levels than Ps-AF patient with 0 risk factor (19.9 ± 3.3 versus 11.9 ± 0.9 ng/mL, p < 0.0001).

### Heart Failure

In the subgroup of patients without heart failure (N = 123), after multivariable analyses, Gal-3 level (HR = 1.11 [1.02–1.20] per unit increase, p = 0.02) and LAD (HR = 1.08 [1.03–1.13] per unit increase, p = 0.002) were also the only independent predictor of recurrences.

### Arrhythmia-free Survival

**Figure 1:**
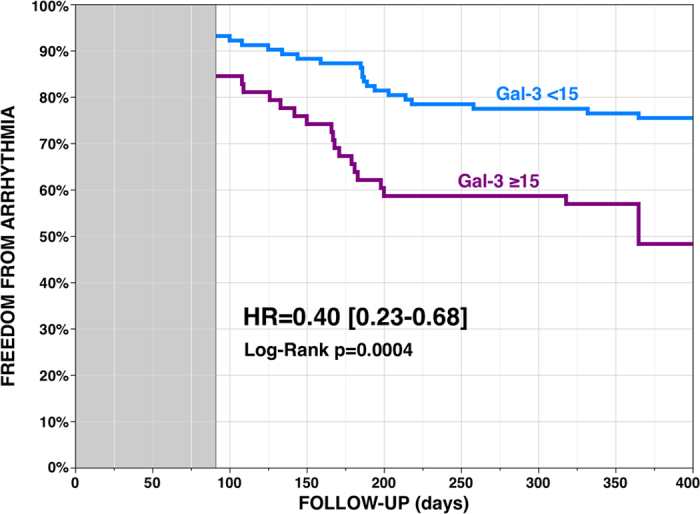
Arrhythmia-free survival without anti-arrhythmic drug after a single ablation procedure according to galectin-3 baseline serum level, <15 (N = 102, 64%) or ≥15 ng/mL (3-month blanking period).

**Figure 2:**
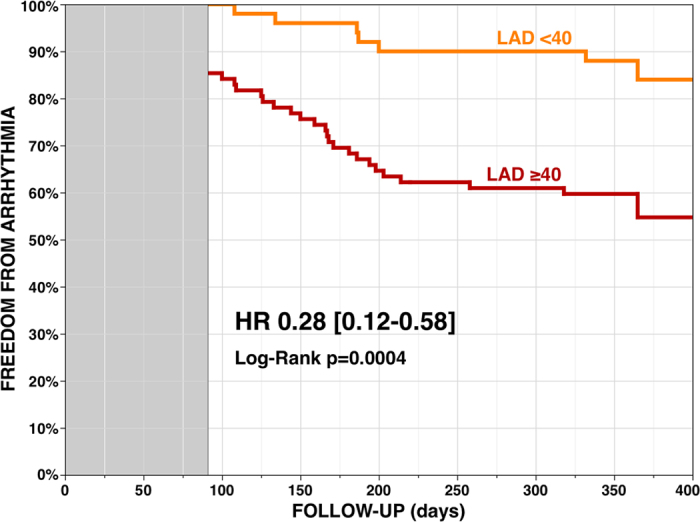
Arrhythmia-free survival without anti-arrhythmic drug after a single ablation procedure according to left atrial diameter, <40 (N = 62, 39%) or ≥40 millimeters (3-month blanking period).

Kaplan-Meier analyses showed a 61% reduction of the risk of recurrence at 12 months after a single procedure in patients with Px-AF (N = 88, 55%), as compared with Ps-AF patients. Gal-3 level identified a group at lower risk of recurrence, whatever the type of AF: a 60% reduction in patients with baseline Gal-3 <15 (N = 102, 64%) with a 12-month recurrence rate of 25% versus 52% in patients with Gal-3 ≥15 (p = 0.0005) (Fig. 1). LAD <40 (N = 62, 39%) also identified patients at lower risk of recurrence (Fig. 2).

**Figure 3:**
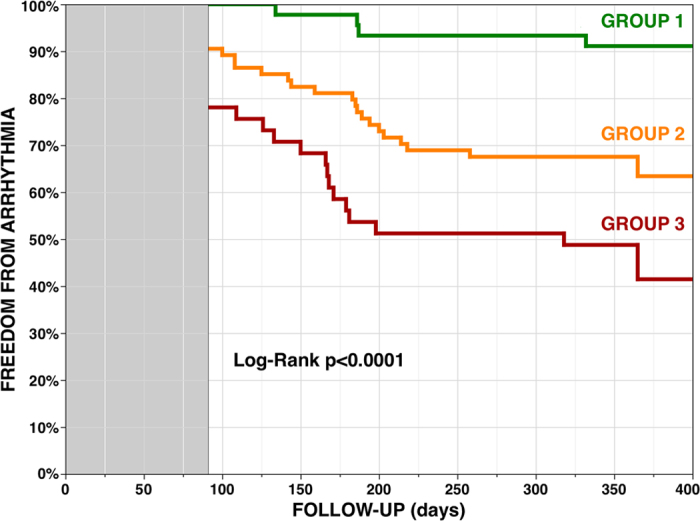
Arrhythmia-free survival without anti-arrhythmic drug after a single ablation procedure in patients with galectin-3 level <15 ng/mL and a left atrial diameter <40 millimeters (Group 1, 28%), either galectin-3 level ≥15 or a left atrial diameter ≥40 (Group 2, 46%), or both galectin-3 level ≥15 and a left atrial diameter ≥40 (Group 3, 26%) (3-month blanking period).

Forty-five patients (28%) had a Gal-3 level <15 and an LAD <40 (Group 1, 87% with Px-AF), 74 (46%) had either a Gal-3 level ≥15 or an LAD ≥40 (Group 2, 50% with Px-AF), and 41 (26%) had both a Gal-3 level ≥15 and an LAD ≥40 (Group 3, 29% with Px-AF). Patients in Group 1 were at low risk with 91% of patients free of arrhythmia at 1 year, following a single procedure, and without any anti-arrhythmic drugs; patients in Group 2 were at intermediate risk with 64% of patients free of arrhythmia; patients in Group 3 were at high risk of recurrence with only 41% of patients free of arrhythmia at 1 year (log-rank p < 0.0001) (Fig. 3).

In Px-AF patients, rates of recurrence at 1 year were 8%, 32%, and 42% for patients in Groups 1, 2, and 3, respectively. In Ps-AF patients the rates of recurrence were 17%, 41%, and 66% for patients in Groups 1, 2, and 3, respectively.

## Discussion

### Major Findings

This study showed that: (1) Gal-3 levels and LAD are strong independent predictors of recurrence after AF ablation, whatever the type of AF (paroxysmal or persistent); (2) when combined, Gal-3 levels and LAD identify patients at low, intermediate and high risk of arrhythmia recurrence at 1 year, whatever the type of AF.

### Galectin-3 and Ablation

Gal-3 is highly expressed in fibrotic tissues, and upregulated in chronic inflammatory and fibrotic conditions in human. Gal-3 levels are higher in patients with Ps-AF^5^. Kornej and colleagues in a study of 105 patients (49% with Px-AF) having undergone AF ablation found no association between Gal-3 levels and sinus rhythm maintenance^9^. In that study, levels of Gal-3 were almost twice lower than in ours (7.8 ± 2.9 versus 14.4 ± 5.6), with no difference between Px-AF and Ps-AF patients, as opposed to previously published data^10^. Although it might be explained by usage of different assays, it may also be related to the clinical profile of patients included in that study, with less severe atrial remodeling: 71% of those patients had lone AF, which could explain why the team failed to associate Gal-3 levels with outcomes. Data on rates of freedom from arrhythmia are also lacking. Conversely, Wu and colleagues found in a small cohort of 50 patients with Ps-AF that Gal-3 concentration and LA diameter were independent predictors of AF recurrence after ablation^11^. Our study confirms these data in a cohort three times as large, and with a follow-up twice as long.

### Atrial Remodeling

We identified only 2 independent predictive factors of recurrence, and both are linked with structural atrial remodeling: LAD (a better predictor than LA volume) and Gal-3 level. LAD is already known to be a strong predictor of recurrences after AF ablation, but it was thought to be mainly related to the parallel progression of fibrosis and LA enlargement^12^. However, the distinct role of these 2 parameters, as two independent predictors of recurrence in our study, is confirmed by the results of the DECAAF study in which the extent of fibrosis, assessed with MRI and dedicated software, was not correlated to LA volume^13^. LA enlargement and Gal-3 may then favor AF perpetuation through distinct potentiating mechanisms of atrial remodeling.

The DECAAF study displayed a similar prognostic role of fibrosis after AF ablation. However, MRI lacks adapted availability in regular practice and feasibility. In the DECAAF study, estimation of atrial tissue fibrosis was impossible in 17% of patients because of poor imaging quality. MRI would appear to be a cost ineffective technique as compared to simple measurements of Gal-3 serum level with fully-automated reliable immunoassays. Finally, there is no evidence that either low endocardial voltage or the presence of late gadolinium enhancement reflect actual histological fibrosis within LA, whereas Gal-3 level is pathophysiologically directly related to actual fibrotic tissue activity^14^.

### Remodeling rather than Clinical Presentation

We have shown that patients with Px-AF and 1 or 2 risk factors had a higher risk of recurrence than patients with Ps-AF and no risk factor. The presence or absence of arrhythmogenic substrate rather than the clinical natural history of AF could be related to prognosis after ablation. Indeed, in our study Ps-AF patients without any risk factor had an 83% rate of freedom from arrhythmia at 12 months, after a single procedure, and without any anti-arrhythmic drug, as compared with a 58% rate in Px-AF patients with both risk factors. Markers of atrial remodeling also have the advantage of ignoring the initial cause of the arrhythmogenic substrate. Clinical presentation differs between patients with ageing-related, cardiovascular disease-induced, or AF-induced remodeling substrate, but the results of AF ablation may only be related to the amount of substrate, not its origin. Some authors have suggested that higher Gal-3 levels in AF patients could be related to higher body mass index, hypertension, or diabetes, associated through the metabolic syndrome, rather than to actual atrial remodeling^15^. Metabolic syndrome is known to have a negative impact on the prognosis of patients after AF ablation^16^. The role of these metabolic conditions may be to promote a progression of fibrosis, the Gal-3 level being the end result of multiple metabolic conditions related to AF perpetuation. Gal-3 level may then reflect the actual prognostic arrhythmogenic substrate, no matter what prompted it in the first place^17^. The results of the STAR-AF II study, which found similar results between simple and more extensive ablation strategies in Ps-AF patients, might be partly explained by the fact that an extensive ablation may not be required, or may even be deleterious, in the absence of a significant atrial arrhythmogenic substrate^18^. We show that some Ps-AF patients have low levels of Gal-3, and may thus have limited substrate to target for ablation. Gal-3 level appears to be a better predictor of recurrence in the subgroup of Px-AF patients, and conversely LAD a better predictor in Ps-AF patients. These results might also be explained by the lack of power of subgroup analyses, and need further evaluation.

### Limitations

Patients with HF (23%) were also included in the study despite the bias that Gal-3 levels are higher in that population, and that Gal-3 levels might reflect rather the extent of ventricular fibrosis than the actual atrial substrate. However, HF patients may also be good candidates for AF ablation, especially those without underlying structural heart disease. Gal-3 level might then help identify those with a better prognosis after ablation in that subgroup of patients. Moreover, Gal-3 and LAD remained independent predictors of recurrence even after removing HF patients from the population.

Other confounding factors absent from the Cox model may also have accounted for the differences observed in the study.

The arrhythmia-free survival definition (1-year follow-up, single procedure, 3-month blanking period, ECG and Holter-monitoring documentation) may be criticized, as asymptomatic sustained paroxysmal AF episodes may have been missed. However, 7-day ambulatory telemetry monitors are recognized useful tools in detecting asymptomatic AF episodes after ablation^19^. Moreover, asymptomatic recurrences may have only limited interest, as ablation is firstly a treatment of symptoms.

## Conclusions

In patients undergoing atrial fibrillation ablation, higher levels of galectin-3 and larger left atrial diameters, which reflect more extensive left atrial remodeling, independently predict recurrences of arrhythmia, whether the AF is paroxysmal or persistent, and identify patients at low, intermediate, and high risk. These simple markers may be of considerable interest in defining the therapeutic strategy in candidates for atrial fibrillation ablation.

## Additional Information

**How to cite this article**: Clementy, N. *et al.* Serum Galectin-3 Levels Predict Recurrences after Ablation of Atrial Fibrillation. *Sci. Rep.* **6**, 34357; doi: 10.1038/srep34357 (2016).

## Figures and Tables

**Table 1:** Baseline characteristics for all patients, and for patients with and without recurrence of atrial arrhythmia at 1 year.

	All Patients (N = 160)	No Recurrence (N = 105)	Recurrence (N = 55)	*p*
Male gender (%)	114 (71)	75 (71)	39 (71)	0.95
Age (years)	61 ± 10	60 ± 10	64 ± 10	**0.01**
BMI (kg/m^2^)	29 ± 6	28 ± 6	30 ± 6	0.14
Paroxysmal AF (%)	88 (55)	68 (65)	20 (36)	**0.0006**
Heart Failure (%)	37 (23)	19 (18)	18 (33)	**0.04**
Hypertension (%)	79 (49)	44 (42)	35 (64)	**0.009**
Diabetes Mellitus (%)	27 (17)	17 (16)	10 (18)	0.75
TIA or Stroke (%)	7 (4)	4 (4)	3 (5)	0.63
Vascular Atherosclerosis (%)	11 (7)	7 (7)	4 (7)	0.89
CHADS_2_ Score: 0/1/2/>2	37/58/41/24	30/41/22/12	7/17/19/12	**0.03**
CHA_2_DS_2-_VASC Score: 0/1/2/>2	31/45/42/42	26/30/28/21	5/15/14/21	0.07
VKA/NOAC (%)	105 (66)/55 (34)	66 (63)/39 (37)	39 (71)/16 (29)	0.31
RAAS inhibitor (%)	57 (36)	27 (26)	30 (55)	**0.0003**
Betablocker (%)	81 (51)	47 (45)	34 (62)	**0.04**
AA Drug*: 0/Class I/sotalol/amiodarone	17/90/29/93	14/59/13/54	3/31/16/39	**0.04**
LA Diameter (mm)	42 ± 8	40 ± 7	45 ± 9	**0.001**
MRI Indexed LA Volume (mL/m^2^)	65 ± 19	63 ± 17	71 ± 20	**0.02**
LVEF (%)	54 ± 11	54 ± 10	53 ± 12	0.37
Mitral Regurgitation Grade: 0/I/II/III-IV	81/38/37/4	54/29/19/3	27/9/18/1	0.16
MDRD GFR (mL/min/1.73 m^2^)	75 ± 20	76 ± 20	73 ± 21	0.43
Galectin-3 (ng/mL)	14.4 ± 5.6	13.5 ± 4.8	16.1 ± 6.6	**0.01**
QRS width (ms)	96 ± 20	95 ± 19	98 ± 23	0.67
Sinus rhythm at admission (%)	89 (56)	69 (66)	20 (36)	**0.0004**

^*^Antiarrhythmic drug prescribed at the time of ablation and during the blanking period.

**Table 2:** Cox proportional hazard model for recurrence of sustained atrial arrhythmia after a single procedure of AF ablation.

	Univariable Analysis		Multivariable Analyses	
	HR [95% CI]*	*p*	HR [95% CI]*	*p*
Age (years)	1.03 [1.01–1.06]	**0.02**	1.01 [0.97–1.04]	0.66
BMI (kg/m^2^)	1.04 [0.99–1.08]	0.12		
Sex: male	0.97 [0.57–1.84]	0.99		
Type of AF: persistent	2.59 [1.51–4.57]	**0.0005**	1.47 [0.73–2.99]	0.28
Vascular Disease	1.16 [0.35–2.84]	0.78		
Heart failure	1.89 [1.05–3.27]	**0.03**	0.47 [0.20–1.02]	0.06
Hypertension	2.01 [1.17–3.55]	**0.01**	1.73 [0.91–3.36]	0.09
Diabetes	1.07 [0.51–2.04]	0.84		
Galectin-3 (ng/mL)	1.05 [1.01–1.09]	**0.02**	**1.07 [1.01**–**1.12]**	**0.02**
QRS width (ms)	1.00 [0.98–1.02]	0.64		
LA diameter (mm)	1.07 [1.03–1.11]	**0.0002**	**1.07 [1.03**–**1.12]**	**0.001**
LVEF (%)	0.99 [0.96–1.01]	0.25		
MDRD GFR (mL/min/1.73 m^2^)	0.99 [0.98–1.01]	0.43		

^*^HR [95% CI], hazard ratio with 95% confidence interval. HR value is expressed for continuous variables as per-unit increase for regressor.

**Table 3:** C-statistics for Galectin-3 and Left Atrial Diameter at predicting recurrence at 1 year.

	Cutoff	Se	Sp	PPV	NPV	Se - (1 -Sp)	AUC	*p*
Galectin-3 (ng/mL)	≥15	0.56	0.72	0.52	0.76	0.29	0.62	**0.006**
Left Atrial Diameter (mm)	≥40	0.80	0.54	0.47	0.84	0.34	0.68	**0.0003**
Combined Score*	≥1	0.93	0.39	0.44	0.91	0.32	0.72	**<0.0001**
	≥2	0.44	0.84	0.21	0.74	0.27	0.72	**<0.0001**

ROC analyses: Se, sensitivity; Sp, specificity; PPV, positive predictive value; NPV, negative predictive value; AUC, area under curve.

^*^Combined Score (0, 1, or 2) is calculated as the number of the following risk factors: galectin-3 level ≥15 ng/mL; left atrial diameter ≥40 mm.

**Table 4:** Galectin-3 levels (ng/mL) and rates of patients with arrhythmia recurrence at 1 year according to the type of atrial fibrillation and the number of risk factors.

	ALL (N = 160)	Group 1 (N = 45)	Group 2 (N = 74)	Group 3 (N = 41)	*p*^†^
ALL (N = 160)	14.4 ± 5.6	10.6 ± 4.7	13.6 ± 4.7	20.1 ± 5.4	<*0.0001*
	34.4%	8.9%	36.5%	58.5%	<*0.0001*
Px-AF (N = 88)	13.2 ± 5.2	10.5 ± 2.3	13.9 ± 5.8	19.9 ± 3.3	<*0.0001*
	22.7%	7.7%	32.4%	41.7%	*0.006*
Ps-AF (N = 72)	15.9 ± 5.7	11.9 ± 0.9	13.3 ± 3.2	20.2 ± 6.1	<*0.0001*
	48.6%	16.7%	40.5%	65.5%	*0.03*
*p*^‡^	*0.0001*	0.15	0.76	0.46	
	*0.0006*	0.51	0.47	0.16	

Group 1: patients with galectin-3 level <15 ng/mL and a left atrial diameter <40 millimeters; Group 2: patients with either galectin-3 level ≥15 or a left atrial diameter ≥40; Group 3: patients with both galectin-3 level ≥15 and a left atrial diameter ≥40. *p*^†^, p-value for comparison between risk factors groups; *p*^‡^, p-value for comparison between paroxysmal (Px-AF) and persistent (Ps-AF) atrial fibrillation patients.
